# Can we still do something-and what?-for a seemingly missing syndrome?

**DOI:** 10.1186/s13052-019-0621-2

**Published:** 2019-02-28

**Authors:** Rita Campi, Maurizio Bonati

**Affiliations:** Laboratory for Mother and Child Health, Department of Public Health, Istituto di Ricerche Farmacologiche Mario Negri-IRCCS, Via Giuseppe La Masa 19, 20156 Milan, Italy

**Keywords:** Infant mortality, Sudden unexpected deaths in infancy, Sudden infant death syndrome, Italy

## Abstract

**Background:**

Although several modifiable risk factors have been identified, and prone and side sleep positions were identified as preventing sudden infant death syndrome (SIDS), the epidemiology of sudden unexpected deaths in infancy (SUDI), which includes SIDS, has not unchanged in over a decade. What can be done?

**Methods:**

Italian infant mortality rates were analysed between 1996 and 2015.

**Results:**

Between 1996 and 2015 in Italy 1152 SUDI deaths were reported in infants less than one year old. SUDI decreased substantially from 18 in 1996 to 10.2 deaths per hundred live births in 2015 (− 43%), the contribution was the change in the SIDS rate from 11.3 to 4.1 (− 64%). However, since 1004 main and SIDS rates have not changed.

**Conclusion:**

Interventions that support safe sleep must be maintained, but research is still needed since although these dramatic deaths have been reduced their causes remain unknown. The challenge is now to shift their trend which has been constant for too long.

## Background

Over recent decades infant mortality has dropped a dramatically in countries with ample resources because of improvements in public health policy and medical advances [1]. In the past effective programs were made to reduce neonatal mortality, and more recently efforts have focused on prevention to reduce postneonatal mortality, particularly sudden unexpected deaths in infancy (SUDI); this included sudden infant death syndrome (SIDS) among those that cannot be explained after thorough investigation, and these are the leading cause of postneonatal mortality in the developed world. The proven relation between SIDS and prone sleep position, and the subsequent public education and effectiveness of the “Back to Sleep” campaigns were the elements to which the drastic reduction in postneonatal mortality were attributed [2]. However, SUDI deaths are still occurring.

A few potential mechanisms responsible for the intrinsic vulnerability of SIDU infants remain unclear but may be the result of both pre and postnatal environmental conditions and genetically determined mal – development or delay in maturation [3]. Thus, new different action is required to improve our knowledge and plan interventions to reduce infant mortality in developed countries [4]. What is the lasted epidemiological situation? What must we do about it [5]?

## Methods

### Data source

Data include all deaths of resident infants up to one year old in the 20 Italian regions during the period 1996–2015. Overall and cause-specific mortality rates were calculated per 100,000 live births and 95 % confidence intervals (95% CIs) of mortality rates were computed. Data were gathered from ISTAT (National Institute of Statistics) “vital statistics on causes of death” database, http://siqual.istat.it/SIQual/lang.do?language=UK.

Causes of death are classified according the International Classification of Diseases (ICD), ninth revision from 1993 to 2002 and tenth revision from 2003 to 2015. SUDI deaths were classified with the ICD-9 codes 798.0, 799.9 and E913.0 and ICD-10 codes R95, R99 and W75 [2, 3, 6–8]. The number of live births in the period 1996–2015 was used as the denominator for calculating of mortality rates.

### Data analysis

Data are presented using descriptive statistics. A linear sigmoid trend analysis was conducted for SUDI and SIDS to predict total rates (with 95% CIs) over the years 1996–2015, interpolating values using the following formula: Y=Bottom + (Top-Bottom)/(1 + 10^((LogIC50-X)*HillSlope)). Chi-square test was used to determine differences in between groups in the population. Statistical analysis was done with the statistical package Stata Release 9 (StataCorp, Texas, USA, 2005) and PRISM version 5.03 (GraphPad Software). Because this investigation used publicly available anonymous data, no ethics committee approval was required.

## Results

After the dramatic falls in infant mortality rates in the twentieth century, from 1996 to 2015 the decline continued, from 618 to 310 per hundred thousand Italian live births (50% decrease). The main contribution to the overall change is the decrease in early neonatal mortality (58%), rather than to late neonatal mortality (44%) and postneonatal mortality (37%). In 2015 early neonatal mortality, late neonatal mortality, and postneonatal mortality accounted for 47, 21, and 32% of Italian infant mortality, respectively.

**Fig. 1:**
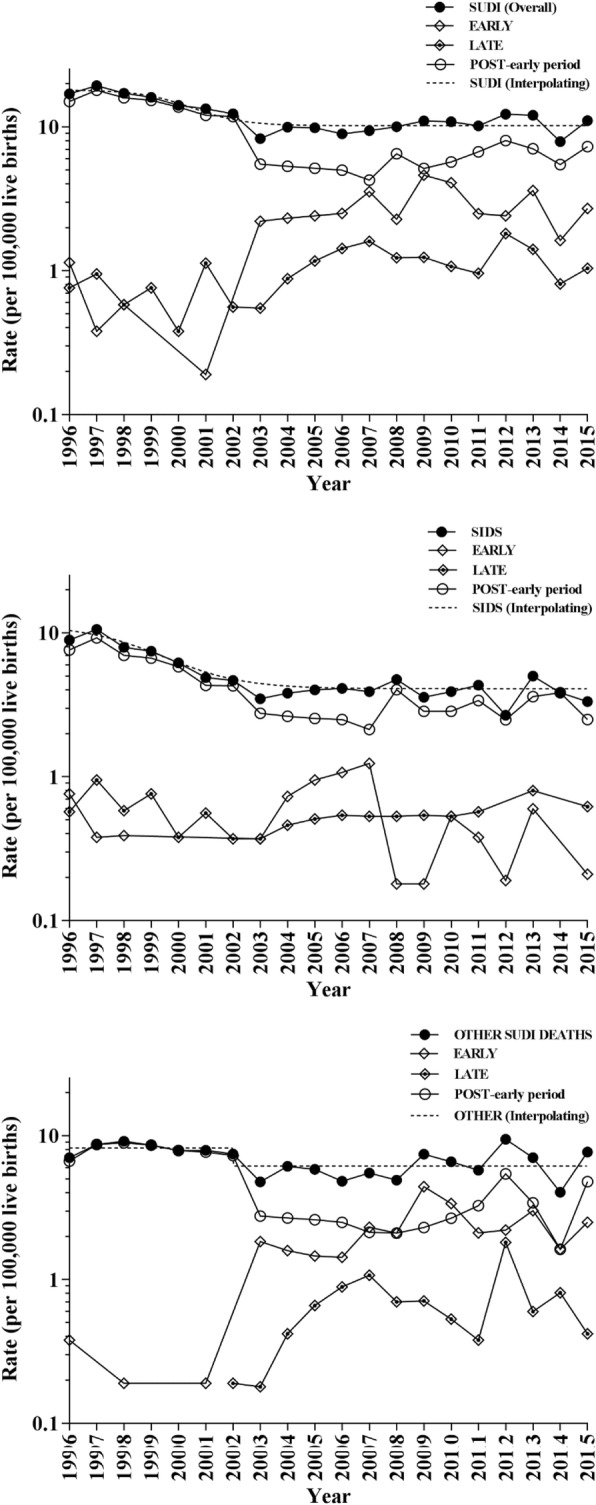
SUDI (sudden unexpected death in infancy) overall mortality rates (early neonatal, late neonatal, and postneonatal), SIDS (sudden infant death syndrome), and other SUDI causes of death in Italy, 1996–2015

Perinatal problems, congenital anomalies, and SUDI are again the three main causes of infant mortality (73.1, 20.2, and 2.4% in 2015). Perinatal problems are the first cause of death during both early and late neonatal periods, while congenital malformations are the first cause in the post-neonatal period. The ranks were maintained over the 20-year period analysed. Although perinatal problems and congenital anomalies mortality fell more than 50%, SUDI rates dropped only 35% from 1996 to 2015. Between 1996 and 2015 in Italy 1152 SUDI deaths were reported in infants less than 1 year of age; 670 (57.3%) deaths were males, 888 (77.1%) in the postneonatal period, and 510 (44.3%) were classified as due to SIDS. SUDI trends indicated an overall rate decrease from 18 deaths per hundred thousand live births in 1996 to 10.2 deaths per hundred thousand live births in 2015 – a reduction of 43.3% over 20 years (Fig. 1). Stratified analyses in SUDI trends by SIDS and other classified causes of death indicated the greatest decrease in the SIDS rate was from 11.3 (9.6 – 13, 95% CI) deaths per hundred thousand live births in 1996 to 4.1 (3.6 – 4.6) per hundred thousand live births in 2015 – a reduction of 63.7% compared to 24,4% for other SUDI causes of death (from 8,2 to 6.2 deaths per hundred thousand live births). The year when the rate was halved was 2000 for SUDI and 1999 for SIDS. According to the model (R square 0.9289 and 0.8817) since 2004 infant mortality rates for both SIDS and SUDI have not changed: 4.1 deaths per hundred thousand live births (3.6–4.6, 95% CI) and 10.2 (9.4–11.0), respectively, were the estimated values.

With the limits of the sample size, the results of the stratify analysis of deaths by geographic national areas indicated that SUDI overall rates for the considered period were higher in the South (7.2 deaths per hundred thousand live births; 6.6–7.8, 95% CI) than in the North (5.7; 5.2–6.2) or in the Center of Italy (3.3; 2.7–3.9); Chi-square 61.2, *p* < 0.0001.

## Discussion

After the first national campaign, in The Netherlands in 1987, “Back to sleep” initiatives encouraging parents to place their infant to sleep supine spread throughout the north of the world during the 1990s. The effectiveness in reducing sudden infant deaths was attributed to this simple and inexpensive attitude. This happened in many countries, in Italy too, [8] as described here for the first time in detail over a long period. There have been impressive reductions in SIDS and other sudden unexpected deaths in infancy around the world, and rates have been stabilized in many countries (e.i, Netherlands, Ireland, UK, Norway, Sweden, Italy), although with some difference in rates and trends within and between country [4]. Can we reduce them further – and how [5]? This is the question that professionals with different skills now have to strive to respond collaboratively in real practice in the near future. Several modifiable risk factors have been identified, such as maternal smoking in pregnancy, infant over-heating, and soft bedding, although they have still not received enough attention, in all national areas and to maintain over time, as done only in part for the promotion to underpin the critical link with infant sleep position. Information, education and advocacy initiatives must be maintained in order not to lose what has been gained. However, though this reflection is an important public health question the main challenge is basic biological research – on genetic mutations, molecular/control mechanisms, etc. – since the causes of these sudden deaths are not known.

## Conclusions

To further reduce SIDU and SIDS, research into the pathophysiological etiology should have priority over descriptive associations, and over it will all take time. For now, besides ensuring all parents are aware of understand, and counteract risk factors for SIDU and SIDS, particularly the high-risk population, appropriate comfort for bereaved parents must emphasise that the tragic deaths were the result of a natural condition, with no blame. All this must be done even if it is not sufficient on its own to eliminate SIDU and SIDS.
